# Characteristics and Risk Factors of Children Requiring Prolonged Mechanical Ventilation vs. Non-prolonged Mechanical Ventilation in the PICU: A Prospective Single-Center Study

**DOI:** 10.3389/fped.2022.830075

**Published:** 2022-02-08

**Authors:** Yanling Liu, Qingyue Wang, Jun Hu, Fang Zhou, Chengjun Liu, Jing Li, Yueqiang Fu, Hongxing Dang

**Affiliations:** ^1^Department of Pediatric Intensive Care Unit, Ministry of Education Key Laboratory of Child Development and Disorders, Children's Hospital of Chongqing Medical University, Chongqing, China; ^2^National Clinical Research Center for Child Health and Disorders, China International Science and Technology Cooperation Base of Child Development and Critical Disorders, Chongqing, China; ^3^Chongqing Key Laboratory of Child Health and Nutrition, Chongqing, China

**Keywords:** mechanical ventilation, ventilator weaning, intensive care units, pediatric, characteristics, risk factors

## Abstract

**Background:**

Prolonged mechanical ventilation (PMV) has become an enormous challenge in intensive care units (ICUs) around the world. Patients treated with PMV are generally in poor health. These patients represent a select cohort with significant morbidity, mortality, and resource utilization. The status of children who have undergone PMV in China is unknown. Our goal is to investigate the prevalence and characteristics of pediatric patients with PMV, as well as the risk factors of PMV in the pediatric intensive care unit (PICU).

**Methods:**

The subjects were divided into two groups. The PMV group(MV ≥ 14 days) and the non-PMV group(2 days < MV <14 days). The baseline characteristics, treatments, mortality and other results between the two groups were compared. The risk factors associated with PMV were evaluated using univariate and multivariable analyses.

**Results:**

Of the 382 children enrolled, 127 (33.2%) received prolonged mechanical ventilation. The most common cause of MV in the PMV group was acute lung disease (48.0%), followed by acute circulatory system disease (26.0%), acute neurological disease (15.0%), postoperative monitoring (10.2%), and others (0.8%). Comorbidities were more prevalent among the PMV group (*P* = 0.004). The patients with PMV had a higher rate of premature birth (24.4 vs. 14.1%, *P* = 0.013) and higher PIM3 score at admission [5.6(3.0–9.9) vs. 4.1(1.7–5.5), *P* < 0.001]. The use of inotropes/vasopressors (63.8 vs. 43.1%, *P* < 0.001) was more common in patients with PMV compared with those in the non-PMV group. In the PMV group, the rate of extubation failure (39.4 vs. 6.7%, *P* < 0.001) was higher than the non-PMV group. The median hospital stay [35(23.0–50.0)d vs. 20(14.0–31.0)d, *P* < 0.001], PICU stay [22(15.0–33.0)d vs. 9(6.0–12.0)d, *P* < 0.001], hospitalization costs [¥391,925(263,259–614,471) vs. ¥239,497(158,723–350,620), *P* < 0.001], and mortality after 1-month discharge (22.0 vs. 1.6%, *P* < 0.001) were higher in the PMV group. Multivariate analysis revealed that age <1 year old, a higher PIM3 score at admission, prematurity, the use of inotropes or vasopressors, extubation failure, and ventilator mode on the first day of MV were associated with PMV.

**Conclusions:**

The incidence and mortality of PMV in pediatric patients is surprisingly high. Premature infants or patients with severe disease or extubation failure are at higher risk of PMV. Patients with PMV exhibit a greater burden with regard to medical costs than those on non-PMV. It is important to establish specialized weaning units for mechanically ventilated patients with stable conditions.

## Introduction

With the widespread application of mechanical ventilation technology, the survival rate of critically ill patients is constantly increasing. In recent years, numerous studies evaluating the duration of mechanical ventilation of patients with critical illnesses have shown a rapid growth in the number of patients receiving prolonged mechanical ventilation (PMV) (1–4). In China, it is estimated that 36.1% of critically ill patients received ventilation for more than 21 days in adult intensive care units (ICUs) in 2016 (3). After a critical illness, long-term dependence on a ventilator and difficulty in weaning have become new public hygienic challenges.

Prolonging the duration of mechanical ventilation causes many challenging complications, such as ventilator-associated pneumonia (VAP), pulmonary hemorrhage, tracheal injury, diaphragm atrophy, neuromuscular disorders, an increased length of ICU stay and an unfavorable discharge destination (5–8). Moreover, there are no specialized weaning units for patients requiring PMV. Most children with PMV stay in the hospital indefinitely and are separated from their families, which adversely affects children's physical and emotional development. Significant medical resources are devoted to these children, and their families undertake high costs during Pediatric Intensive Care Unit (PICU) stays (6). Consequently, it is necessary to attempt the early weaning of these children. Society and the patient's family must consider the prognosis and quality of life of children who ultimately survive.

Due to a lack of data regarding children with PMV in China, we carried out a prospective cohort study on the pediatric PMV in the PICU. Our objectives were as follows: (1) to understand the incidence and mortality of PMV in the PICU, (2) to identify the risk factors for PMV, and (3) to explore the outcomes of patients on PMV. We hope to provide a reference for the management of children with PMV based on the obtained results.

## Subjects and Methods

### Setting

This prospective cohort study was conducted in the PICU of a tertiary first-class hospital affiliated to a university, with 84 beds, 29 physicians, and 138 nurses on the shift. All patients on ventilation admitted to PICU between October 1, 2020 and June 30, 2021 were screened every day.

### Subjects

The subjects included patients aged 29 days to 18 years old who were ventilated longer than 2 days (more than 6 h per day), including patients on invasive mechanical ventilation (In-MV), non-invasive mechanical ventilation (NIV), and continuous positive airway pressure (CPAP) duration. Those who had a short interruption (<2 days) of ventilation during the weaning process during the same episode of ventilation were included. Patients younger than 29 days and older than 18 years old at admission, or brain-dead were excluded. The criteria of brain death refer to the Determination of Brain Death/Death by Neurologic Criteria, published in JAMA, 2020 (9).

### Definition

Indications for MV in pediatric patients include 4 categories: (1) inadequate oxygenation, (2) inadequate ventilation, (3) need for airway protection, or (4) circulatory failure, in which sedation and MV can decrease the oxygen consumption during breathing (10). There is no unified definition of prolonged mechanical ventilation in children (11). In this study, according to the definition of PMV for adults and the study of Can FK et al. (12, 13) PMV was defined as a duration of mechanical ventilation not <14 days and more than 6 h/d. A ventilation time between 2 and 14 days was defined as non-prolonged mechanical ventilation (non-PMV) to exclude the effect of postoperative anesthesia on breathing (14). In the non-PMV group, patients who died after giving up treatment were also excluded because the duration of MV might have been prolonged if treatment were continued. In-MV can be administered through an endotracheal tube or a tracheostomy tube. NIV and CPAP can be managed through nasal, full-face, total facial mask, or helmet ventilation. A patient is considered for weaning from the ventilator after meeting all of the following criteria: (1) hemodynamics of patient are stable (absence of shock or requirement for pressors or significant arrhythmias); (2) the disease process that led to mechanical ventilation has resolved or improved; (3) the patient is oxygenated adequately (fraction of inspired oxygen <50% and/or low PEEP requirements); (4) the patient is capable of spontaneous breathing and airway protection (no serious disturbance of consciousness, spontaneous cough and expectoration) (15). A unified weaning process was used for all subjects. Questions about the weaning procedure were encouraged and answered by the authors (Yueqiang Fu and Hongxing Dang) at any time before and during the study. Weaning failure was defined as the need for ventilator support within 2 days of evacuating the ventilator. The need for reintubation within 2 days of extubation was viewed as extubation failure.

### Data Collected

Anonymous raw data from patient's medical records, including gender, age, weight, preterm birth, admission category, Pediatric Index of Mortality 3 score (PIM3 score) on admission, Pediatric Logistic Organ Dysfunction 2 score (PELOD2 score) when the patient was enrolled, the department where the patient stayed before being transferred to the PICU, the main diagnosis, the cause of ventilation, and underlying chronic disease. Treatment information (blood transfusion, hemodialysis, vasopressor and sedative analgesics, the duration of ventilation, and the mode of mechanical ventilation) and outcomes were collected on the day from the ventilation started until the patient was discharged. All patients were followed for 1 month after discharge.

### Outcomes

The main outcomes were the incidence and mortality of PMV. The secondary outcomes were the length of stay and hospitalization costs in the PICU, failed extubation, and complications associated with ventilation, including ventilator-associated pneumonia (VAP), pneumothorax, pneumomediastinum, pulmonary hemorrhage, and others (subcutaneous emphysema, airway granulation tissue hyperplasia).

### Statistical Analyses

Descriptive statistics were used for the demographic and medical characteristics of the subjects. The Kolmogorov–Smirnov single sample test was used to test whether the continuous variables were normally distributed. Continuous variables with a normal distribution were tested by the *t*-test, reported as mean ± standard deviation (SD). Data with a non-distribution were tested by the Mann–Whitney U-test, described as the median with the 25 and 75% interquartile range (IQR). Categorical variables were compared using the chi-squared test with counts and percentages. Univariate and multivariable analyses by binary logistic regressions were used to obtain the risk factors of PMV. Collinearity diagnosis was used to assess whether there was multicollinearity between the variables. A variance inflation factor (VIF) between 0 and 5 indicated that there was no collinearity. A *P*-value of 0.05 was considered statistically significant for all tests. Statistical analyses were completed using SPSS 26.0.

### Ethics Review

The study was approved by the review committee of Children's Hospital of Chongqing Medical University in China and registered in the Chinese Clinical Trial Registry (Registration Number: ChiCTR2100045727). Informed consent was obtained by the parents of all enrolled patients.

## Results

**Figure 1:**
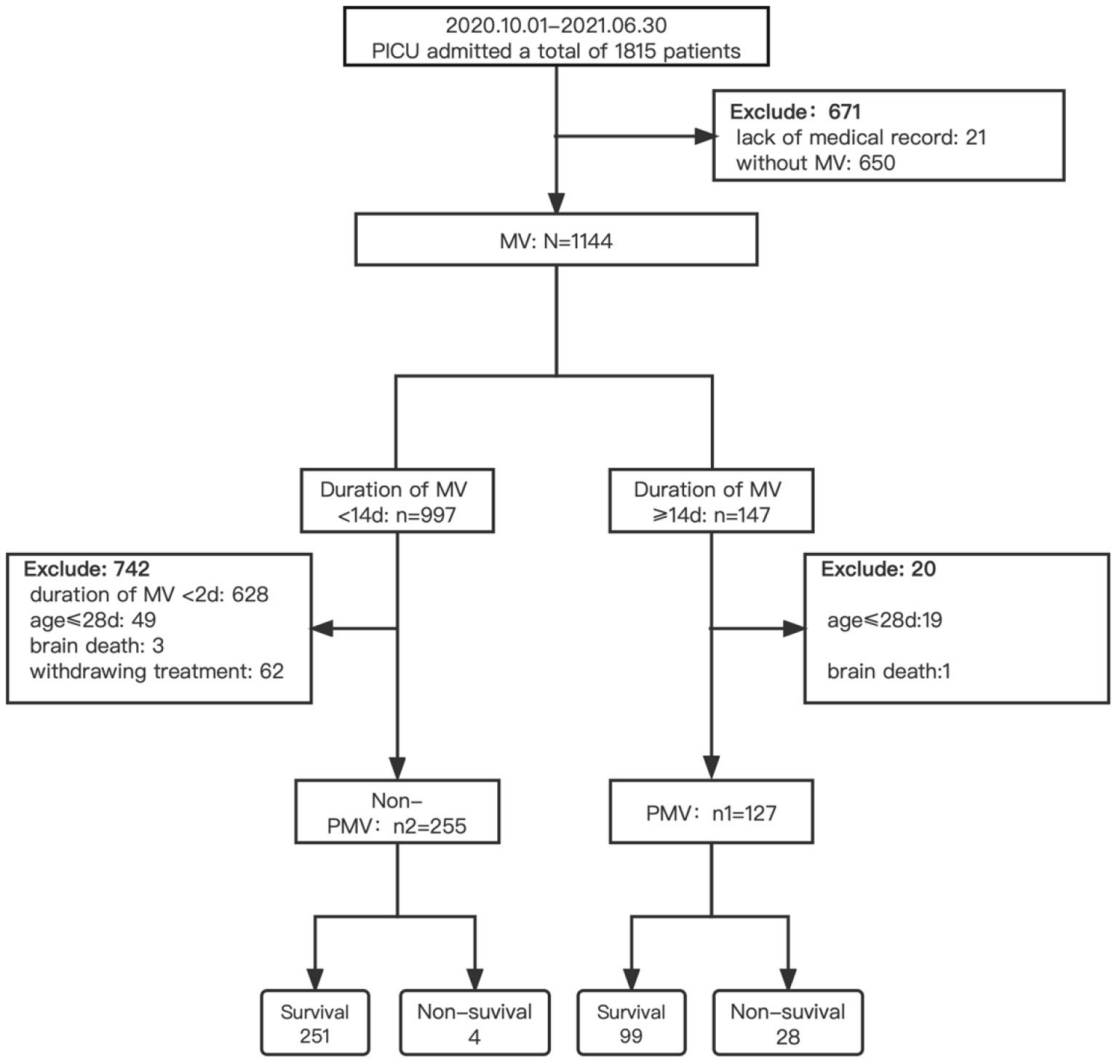
Flow chart of screening. PICU, pediatric intensive care unit; MV, mechanical ventilation; non-PMV, non-prolonged mechanical ventilation; PMV, prolonged mechanical ventilation.

We screened a total of 1,815 patients during the study period. Of 1,144 patients (63.0%) on MV, 382 met the inclusion criteria, of whom 127 (33.2%) received PMV and 255 (66.8%) were treated with non-PMV (Figure 1).

### Demographic and Baseline Data Characteristics

The characteristics of the demographic and baseline data are shown in Table 1. Compared with non-PMV patients, PMV patients were characterized as younger, with a lower weight on admission (*P* < 0.001). There were more patients in the PMV group aged from 29 days to 12 months than the non-PMV group, as shown in Table 2 (65.4 vs. 49.4%, *P* = 0.003). The PIM3 score in the PMV group was profoundly higher than in the non-PMV group. In total, 76.4% patients in the PMV group were admitted to the PICU due to respiratory distress/insufficiency secondary to acute pulmonary diseases, while 53.7% were admitted in the non-PMV group (*P* < 0.001). The incidence of respiratory distress/insufficiency and hemodynamic instability/shock/arrhythmia in the PMV group were higher than the non-PMV group (76.4 vs. 51.0%, 31.5 vs. 15.3%, respectively, *P* < 0.001). Comorbidities were more prevalent among the PMV group. There was no statistical difference in the gender and PELOD2 score between the two groups.

**Table 1:** Demographic and baseline data characteristics.

	**PMV(*n_1_* = 127)**	**Non-PMV(*n_2_* = 255)**	***X*^2^/Z**	***P-*value**
**Gender**, ***n*****%**			0.167	0.683
Male	75 (59.1)	145.0 (56.9)		
Female	52 (40.9)	110.0 (43.1)		
Age, median and inquartile, m	6.0 (2.6–23.0)	12.0 (4.2–47.0)	−3.394	<0.001
Weight, median and inquartile, kg	6.1 (4.2–11.0)	9.0 (6.0–15.0)	−4.458	<0.001
PIM3 score, median and inquartile, %	5.6 (3.0–9.9)	4.1 (1.7–5.5)	−4.531	<0.001
PELOD2 score, mean ± SD	4.0 (3.0–5.0)	4.0 (3.0–5.0)	−0.735	0.463
Preterm birth, *n*%	31 (24.4)	36 (14.1)	6.209	0.013
**Admission category**, ***n*****%**				
Medical, scheduled	13 (10.2)	5 (2.0)	12.930	<0.001
Medical, unscheduled	83 (65.4)	180 (71.1)	1.331	0.249
Surgical, scheduled	9 (7.1)	40 (15.7)	5.607	0.018
Surgical, unscheduled	22 (17.3)	30 (11.8)	2.227	0.136
**Admission origin prior to this PICU admission**, ***n*****%**				
Operational Room in the same hospital	7 (5.5)	71 (27.8)	26.018	<0.001
Emergency Department	12 (9.4)	52 (20.4)	7.279	0.007
General ward in the different hospital	12 (9.4)	41 (16.1)	3.118	0.077
General ward in the same hospital	36 (28.3)	58 (22.8)	1.384	0.239
ICU, PICU or NICU	60 (47.2)	33 (12.9)	54.161	<0.001
**Reason(s) for this PICU admission^∮^, *n*%**				
Respiratory distress / insufficiency	97 (76.4)	130 (51.0)	22.680	<0.001
Hemodynamic instability / Shock / Arrhythmia	40 (31.5)	39 (15.3)	13.567	<0.001
Altered level of consciousness / Seizures /Neurologic problems	8 (6.3)	35 (13.7)	4.680	0.031
Surveillance post-surgery or procedure	28 (22.0)	78 (30.6)	3.085	0.079
Metabolic disorders	5 (3.9)	3 (1.2)	1.948	0.163^&^
Trauma / Burns	1 (0.8)	10 (3.9)	1.962	0.161^&^
Others^Δ^	3 (2.4)	1 (0.4)	/	0.109*
**Main diagnosis**, ***n*****%**				
Acute Pulmonary	61 (48.0)	107 (42.0)	1.268	0.260
Acute Cardiac	33 (26.0)	26 (10.2)	16.181	<0.001
Post-Surgery	13 (10.2)	75 (29.4)	17.582	<0.001
Acute Neurologic	19 (15.0)	41 (16.1)	0.080	0.778
Others^§^	1 (0.8)	6 (2.4)	/	0.432*
**Underlying chronic disease(s) ^∯^, *n*%**				
Congenital heart defects	71 (55.9)	102 (40.0)	8.656	0.003
Acquired heart diseases	64 (50.4)	105 (41.2)	2.920	0.088
History of Cardiac surgery	7 (5.5)	3 (1.2)	4.665^&^	0.031
Chronic pulmonary disease	43 (33.9)	52 (20.4)	8.228	0.004
Neuromuscular disease	12 (9.4)	15 (5.9)	1.642	0.200
Oncologic disease	8 (6.3)	11 (4.3)	0.707	0.400
Organ transplant	4 (3.1)	2 (0.8)	/	0.098*
Hypoalbuminemia, *n*%	68 (53.5)	128 (50.2)	0.380	0.537

PIM3, Pediatric Index of Mortality 3; PELOD2, Pediatric Logistic Organ Dysfunction 2; PICU, Pediatric Intensive Care Unit; ICU, Intensive Care Unit; NICU, Neonatal Intensive Care Unit. Reason(s) for this PICU admission^∮^, a patient may be admitted to PICU for multiple reasons. Underlying Chronic Disease(s) ^∯^, a patient may have multiple underlying diseases. Others^Δ^, 2 cases of chronic renal failure, 1 each of severe immune deficiency, bone marrow suppression after chemotherapy, organophosphorus poisoning. Acute pulmonary, Pneumonia, Bronchiolitis, Asthma, Pulmonary aspiration, Pulmonary edema / effusion, Upper airway obstruction, Chest trauma, Pulmonary dysfunction post-surgery or trauma, Apnea/Respiratory arrest; Acute Cardiac, Pulmonary hypertension, Arrhythmia, Cardiac arrest, Heart failure, Shock; Post-surgery, Cardiac surgery, Thoracic surgery, Neurosurgery, Orthopedic surgery, Craniofacial / ORL surgery, Abdominal surgery; Acute neurologic, Traumatic brain injury, Non-traumatic CNS bleed, Seizures, CNS infections, Acute neuromuscular disease; Others^§^, Trauma other than CNS, Burns, Sepsis. p^*^ used by Fisher Exact test; P^&^ computed by Continuity Correction.

**Table 2:** The number of patients in different age.

**Age**	**PMV (*n_1_*= 127)**	**Non-PMV (*n_2_* = 255)**	***X* ^2^**	***P-*value**
29d−12m	83 (65.4)	126 (49.4)	8.696	0.003
1–3y	20 (15.7)	54 (21.2)	1.599	0.206
3–6y	9 (7.1)	31 (12.2)	2.325	0.127
6–18y	15 (11.8)	44 (17.3)	1.924	0.165

### Treatment Information and Outcomes

In the PMV group, more patients used vasopressors or inotropics than the non-PMV group (63.8 vs. 43.1%, *P* < 0.001). There was no statistical difference in the intermittent use of sedative and analgesic drugs or respiratory medications other than systemic steroids between the two groups. More patients underwent blood transfusion in the PMV group than the non-PMV group (80.3 vs. 55.3%, *P* < 0.001). In the two groups, more patients underwent In-MV compared with NIV on the first day of MV in Table 3. The rate of extubation failure in the PMV group was much higher than in the non-PMV group (*P* < 0.001).

**Table 3:** Treatment information and outcomes.

**Treatment**	**PMV(*n_1_* = 127)**	**Non-PMV(*n_2_* = 255)**	***X*^2^/Z**	***P-*value**
Use inotropes/vasopressors, *n*%	81 (63.8)	110 (43.1)	14.450	<0.001
Continuous infusion of sedatives and analgesics, *n*%	123 (96.9)	255 (100.0)	/	0.012*
Intermittent doses of sedatives and analgesics, *n*%	126 (99.2)	253 (99.2)	/	1.000*
Respiratory Medications^∮^, *n*%				
Systemic Steroid	81 (63.8)	189 (74.1)	4.372	0.037
Inhaled Steroid	124 (97.6)	251 (98.4)	0.297	0.586
Inhaled Beta Agonist	82 (64.6)	169 (66.3)	0.110	0.740
Inhale Anticholinergic Agent	64 (50.4)	137 (53.7)	0.377	0.539
Blood transfusion, *n*%	102 (80.3)	141 (55.3)	22.929	<0.001
Hemodialysis, *n*%	22 (17.3)	21 (8.2)	7.009	0.008
Duration of ventilation, median and inquartile, d	22 (16.0–32.0)	6 (4.0–9.0)	−15.964	<0.001
CPAP within 2 days of MV, *n*%	27 (21.3)	30 (11.8)	6.021	0.014
At least once extubation failure, *n*%	50 (39.4)	17 (6.7)	62.691	<0.001
Ventilator mode on the first day of MV, *n*%			19.298	<0.001
Invasive MV	99 (78.0)	238 (93.3)		
Non-invasive MV	28 (22.0)	17 (6.7)		
Outcomes				
Mortality after 1m discharge, n%	28 (22.0)	4 (1.6)	46.322	<0.001
Hospital stays, median and inquartile, d	35 (23.0–50.0)	20 (14.0–31.0)	−7.469	<0.001
Days of PICU, median and inquartile, d	22 (15.0–33.0)	9 (6.0–12.0)	−11.350	<0.001
Hospitalization cost, median and inquartile, CNY	391,925 (263,259–614,471)	239,497 (158,723–350,620)	−6.866	<0.001
Tracheotomy, *n*%	9 (7.1)	1 (0.4)	10.419	0.001^&^
Home mechanical ventilation, *n*%	3 (2.4)	0	/	0.036*
With complications associated with MV^∯^, n%	51 (40.2)	75 (29.4)	4.429	0.035
Ventilator-Associated Pneumonia	44 (34.6)	68 (26.7)	2.604	0.107
Pneumothorax	11 (8.7)	7 (2.7)	6.609	0.010
Pneumomediastinum	4 (3.1)	5 (2.0)	0.132	0.716^&^
Pulmonary hemorrhage	3 (2.4)	2 (0.8)	/	0.338*
Others^Δ^	6 (4.7)	3 (1.2)	3.225	0.073^&^

CPAP, Continuous Positive Airway Pressure; MV, Mechanical Ventilation; PICU, Pediatric Intensive Care Unit; Respiratory Medications^∮^, a patient may take several of the following respiratory medications simultaneously; With complications during MV^∯^, a patient may develop one or more complications associated with mechanical ventilation; Others^Δ^, include subcutaneous emphysema, airway granulation tissue hyperplasia. p^*^ used by Fisher Exact test; P^&^ computed by Continuity Correction.

Mortality rates after 1-month discharge in the PMV group were significantly higher than in the non-PMV group (22.0 vs. 1.6%, *P* < 0.001). The median hospital stays and PICU stays were also higher in the PMV group (*P* < 0.001). Patients with PMV were more likely to experience ventilator-related complications than those in the non-PMV group (40.2 vs. 29.4%, *P* = 0.035). The median hospitalization cost of patients with PMV was ¥391,925 (263,259–614,471) CNY, while the patients in the non-PMV group was¥239,497 (158,723–350,620) CNY (*P* < 0.001) in Table 3.

Only a small number of people underwent tracheotomy and home mechanical ventilation. In the PMV group, nine (7.1%) patients underwent tracheotomy, and eight of them underwent tracheotomy after 14 days of continuous mechanical ventilation. Only one patient had a tracheotomy after 3 days of mechanical ventilation before admission. The reason for the tracheotomy was unclear. One patient had a tracheotomy before admission and was successfully withdrawn from the ventilator after 5 days of mechanical ventilation via a tracheal tube. Three patients with PMV received home mechanical ventilation after discharge. One patient diagnosed with spinal muscular atrophy accompanied with respiratory distress continued to ventilate at home after discharge and died of respiratory failure within 1 month of discharge. One patient was diagnosed with obstructive sleep apnea syndrome combined with obesity and adenoid hypertrophy. He continued to undergo night BiPAP-assisted ventilation after discharge and was weaned successfully. Another patient had a posterior fossa medulloblastoma with a central metastasis and remained on ventilator support.

### Regression Analysis to Identify the Risk Factors of PMV

Univariate and multivariable analyses in the binary logistic regression model were performed to compare the patients in the PMV group and non-PMV group. Multivariable analysis indicated that age, receiving MV for pulmonary or cardiac disease, PIM3 score at admission, preterm birth, use of inotropes or vasopressors, extubation failure, ventilator mode on the first day of MV, and days in the PICU were associated with PMV. The VIF, which ranged from 1.035 to 1.161, confirmed no multicollinearity in these risk factors (Table 4).

**Table 4:** Logistic regression models assessing the variables associated with prolonged mechanical ventilation.

	***OR***	***95% CI***	***Sig*.**	***VIF***
**Univariate analysis**				
Gender, female or male^*^	0.914	0.593–1.408	0.683	/
Age, per 1month increase	0.988	0.993–1.002	0.325	/
age <1 year old, yes or no^*^	1.931	1.243–3.000	0.003	/
Weight, per 1 kg increase	0.971	0.949–0.994	0.013	/
PIM3 score, per 1% increase	1.040	1.018–1.062	<0.001	/
PELOD2 score, per 1 increase	1.094	0.975–1.228	0.128	/
Preterm birth, yes or no^*^	1.964	1.148–3.360	0.014	/
Admission category, Medical or Surgical^*^	1.172	0.718–1.912	0.526	/
PICU admission for postoperative management, yes^*^ or no	1.558	0.948–2.561	0.080	/
MV for pulmonary or cardiac disease, yes or no^*^	0.283	0.167–0.481	<0.001	/
Comorbidity, yes or no^*^	2.450	1.332–4.506	0.004	/
Hypoalbuminemia, yes or no^*^	1.144	0.747–1.752	0.538	/
Use inotropes/vasopressors, yes or no^*^	2.321	1.497–3.599	<0.001	/
Systemic Steroid, yes or no^*^	0.615	0.389–0.972	0.037	/
Blood transfusion, yes or no^*^	3.299	1.996–5.451	<0.001	/
Hemodialysis, yes or no^*^	2.335	1.230–4.431	0.009	/
CPAP within 2 days of MV, yes or no^*^	2.025	1.144–3.584	0.015	/
At least once extubation failure, yes or no^*^	9.091	4.953–16.686	<0.001	/
Ventilator mode on the first day of MV, NIV or In-MV^*^	3.960	2.074–7.559	<0.001	/
Hospital stays, per 1day increase	1.051	1.036–1.067	<0.001	/
Days of PICU, per 1day increase	1.189	1.144–1.235	<0.001	/
With ventilator-associated complications during MV, yes or no^*^	1.611	1.032–2.514	0.036	/
**Multivariate analysis**				
age <1 year old, yes or no^*^	4.473	2.060–9.713	<0.001	1.052
PIM3 score, per 1% increase	1.066	1.037–1.095	<0.001	1.078
Preterm birth, yes or no^*^	2.515	1.127–6.070	0.025	1.035
MV for pulmonary or cardiac disease, yes or no^*^	3.791	1.575–9.124	0.003	1.100
Use inotropes/vasopressors, yes or no^*^	2.411	1.196–4.858	0.014	1.068
At least once extubation failure, yes or no^*^	11.969	4.805–29.817	<0.001	1.126
Ventilator mode on the first day of MV, NIV or In-MV^*^	2.785	1.090–7.116	0.032	1.099
Days of PICU, per 1 day increase	1.210	1.151–1.272	<0.001	1.161

* *, Parameter. PIM3 score, Pediatric Index of Mortality 3 score; PELOD2 score, Pediatric Logistic Organ Dysfunction 2 score; PICU, Pediatric Intensive Care Unit; MV, Mechanical Ventilation; CPAP, Continuous Positive Airway Pressure; In-MV, Invasive Mechanical Ventilation; NIV, Non-invasive mechanical Ventilation; OR, Odds Ratio; 95% CI, 95% Confidence Interval; VIF, Variance Inflation Factor, computed by colinear diagnosis in linear regression models*.

## Discussion

In this prospective cohort study, we found a high incidence of critically ill patients requiring PMV (MV support ≥ 14 days) in the PICU (33.2%). Most of patients with PMV were transferred from the intensive care units of other hospitals (including ICUs, PICUs, and NICUs), with mechanical ventilation before admission. The majority of children were younger than 12 months old, with higher PIM3 score at admission. This indicates that the age and severity of disease are highly related to PMV. In addition, the high mortality in the PMV group was also noteworthy. PMV has been reported to be associated with mortality after cardiac surgery (16). Patients with PMV had a longer PICU stay, higher risk of ventilator-related complications, higher costs, and worse outcomes. This creates a vicious cycle between PMV, length of hospital stays and complications, factors which aggravate one an other.

In a survey on PMV in mainland China, the proportion of PMV was 36.1% in adults (3), which is higher than the incidence in children in this study. There is a great difference in the disease spectrum between children with PMV and PMV in adults, for this reason the management of children with PMV is not same as that of adults. Degenerative neuromuscular disease and chronic lung disease (chronic obstructive pulmonary disease) (3) are more common in adults treated with PMV. The most common cause of mechanical ventilation in our study was respiratory distress secondary to acute respiratory diseases, followed by postoperative monitoring, acute circulatory diseases, acute neurological diseases, and others (including sepsis, trauma, or burns). The results are not in line with previous reports due to the disunity of the definition of PMV and regional differences (4, 17–19). As children mainly require mechanical ventilation due to acute respiratory diseases, which are easier to treat than chronic diseases, the incidence of PMV in children is lower than that of adults. However, patients with PMV have more comorbidities than those with non-PMV (20). Congenital heart disease, as the most common comorbidity in patients with PMV, is associated with PMV. The reasons for this are as follows: (1) Children with congenital heart disease are prone to pulmonary infections and require respiratory support. (2) Patients with complex congenital heart disease have longer periods of postoperative monitoring and are prone to polyinfections (18). Therefore, patients on MV with congenital heart disease should be of great concern to clinicians.

Since the 20^th^ century, MV has been applied as a life-support technique for multiple indications (21). In the process, many ventilator-associated complications have been observed, which have had an adverse effect on the prognosis of patients. It is important to shorten the time of mechanical ventilation as much as possible. Early extubation and weaning are conducive to the early activities of critically ill patients, and to reducing ventilator-related complications, PICU stays, and hospital stays (22, 23). The ability to identify patients at high-risk of PMV, as well as the risk factors of PMV, may help modify medical approaches to enable early weaning from the ventilator.

This observation suggests that patient <1 year old, born prematurity, who have undergone the use of inotropics or vasopressors, and non-invasive mechanical ventilation at the beginning of mechanical ventilation, exhibit the risk factors of PMV. In particular, the effects of prematurity and initial mode of MV have not been addressed in previous studies. However, children are constantly growing and developing. MV has a greater impact on children's respiratory and circulatory systems. We found that preterm infants had more than two-times the risk of PMV compared to full-term infants. This may be related to their immature airways and lungs. Premature children have narrow airways, which are more likely to experience airway edema and obstruction. In addition, narrow airways increase the risk of weaning failure during mechanical ventilation. If combined with bronchopulmonary dysplasia, a long-term oxygen supply may be required (24). Patients with hemodynamic instability may need to use inotropics or vasopressors and require mechanical ventilation through the endotracheal tube, which indicates that the condition is more serious. Early weaning is conducive to recovery, but patients are more difficult to wean after repeated extubation failure (25, 26).

In our study, there were few patients who underwent a tracheotomy or at-home mechanical ventilation. The specific reasons for this need to be studied further, and may be influenced by medical expenses, family care, and parents' cognition and acceptance of tracheotomiesy or MV. On account of the lack of specialized weaning or step-down units in China, it is difficult for patients to be transferred to other places to continue mechanical ventilation. Commensurately, the duration of PICU stays and hospitalization costs have increased. Sobotka et al. (22) found that more than half of the delayed discharges of children with prolonged mechanical ventilation were caused by a lack of professional or home care after discharge. Studies conducted in Europe, the US, and Taiwan have shown that the establishment of stepped up nursing units and weaning centers was an advantage to the weaning and nursing care of patients dependent on ventilators and the rational utilization of medical resources (6, 8, 27–30). A study in the United States showed that patients with home mechanical ventilation (HMV) had a lower rate of depression, pressure ulcers, and mortality patients in hospital long-term care (HLTC). In addition, patients with HMV paid one-third of the cost of HLTC for health maintenance at home (22). For applicable patients, HMV is a viable and economical option. Prolonged mechanical ventilation has a great impact on children's physical and mental health. As the duration of mechanical ventilation increases, the risks of ventilator-associated complications and nosocomial infections increase. In addition, patients are unable to communicate with their parents in the PICU. Consequently, their cognitive and psychological development are affected (24).

The results of our study can be applied in clinical practice. Focusing on the most common causes of PMV in children as well as the major risk factors may reduce the incidence of PMV. In addition, we suggest that specialized weaning units should be established for mechanically ventilated patients with stable conditions. HMV should be developed and popularized, so that patients with mechanical ventilation can return to their families and society.

This study has certain limitations. First, being a single-center observational study exclusively conducted in our hospital, it only allowed us to show the local situation with a limited number of participants, and was not representative of major centers of China, such as Beijing and Shanghai, with regard to economic and medical disparity. Nevertheless, to date, there are no reports on the reality of MV in mainland China and Chongqing is also one of the major centers of China, representing the southwest of the country. Despite this, a national multicenter survey with a larger sample size is needed to verify our findings. Second, the socioeconomic characteristics of our population were not included, which may constitute a potential confounding factor, and the duration of study was relatively short. In addition, it is necessary to further explore the needs, as well as the attitudes, of children and patients toward mechanical ventilation at home.

## Data Availability Statement

The datasets presented in this study can be found in online repositories. The names of the repository/repositories and accession number(s) can be found below: https://data.mendeley.com/datasets/ypt4d5h5yd/1.

## Ethics Statement

The studies involving human participants were reviewed and approved by the Review Committee of Children's Hospital of Chongqing Medical University, China. Written informed consent to participate in this study was provided by the participants' legal guardian/next of kin.

## Author Contributions

YL and HD: conceptualization, writing-review, and editing. QW: data curation and investigation. JH and FZ: formal analysis, methodology, software, and writing-original draft. HD: project administration and supervision. JL, CL, and YF: resources, visualization, and validation. All authors contributed to the article and approved the submitted version.

## Funding

Chongqing Science and Health Joint Medical Research Project: 2020FYYX082. Future Medicine Youth Innovation Team Development Project of Chongqing Medical University: Basic and clinical pediatrics of critical care.

## Conflict of Interest

The authors declare that the research was conducted in the absence of any commercial or financial relationships that could be construed as a potential conflict of interest.

## Publisher's Note

All claims expressed in this article are solely those of the authors and do not necessarily represent those of their affiliated organizations, or those of the publisher, the editors and the reviewers. Any product that may be evaluated in this article, or claim that may be made by its manufacturer, is not guaranteed or endorsed by the publisher.
